# Strategy for encoding and comparison of gene expression signatures

**DOI:** 10.1186/gb-2007-8-7-r133

**Published:** 2007-07-05

**Authors:** Yajun Yi, Chun Li, Clay Miller, Alfred L George

**Affiliations:** 1 Department of Medicine, Garland Avenue, Vanderbilt University, Nashville, Tennessee 37232-0275,USA; 2 Department of Biostatistics, Garland Avenue, Vanderbilt University, Nashville, Tennessee 37232-0275,USA; 3 Department of Pharmacology, Garland Avenue, Vanderbilt University, Nashville, Tennessee 37232-0275,USA

## Abstract

EXALT (EXpression signature AnaLysis Tool) is a computational system enabling comparisons of microarray data across experimental platforms and different laboratories . An essential feature of EXALT is a database holding thousands of gene expression signatures extracted from the Gene Expression Omnibus, and encoded in a searchable format. This novel approach to performing global comparisons of shared microarray data may have enormous value when coupled directly with a shared data repository.

## Abstract 2

EXALT (EXpression signature AnaLysis Tool) enables comparisons of microarray data across experimental platforms and different laboratories.

## Rationale

The application of high-throughput microarray technology for determining global changes in gene expression is an important and revolutionary experimental paradigm that facilitates advances in functional genomics and systems biology. Widespread use of this approach is evident in the rapid growth of microarray datasets stored in public repositories [1,2]. For example, the Gene Expression Omnibus (GEO), curated by the National Center for Biotechnology Information (NCBI), has received thousands of data submissions representing more than 3 billion individual molecular abundance measurements [3,4].

The growth in microarray data deposition is reminiscent of the early days of GenBank, when exponential increases in publicly accessible nucleotide sequence data occurred. However, unlike nucleotide sequences, microarray datasets are not as easily shared by the research community, resulting in many investigators being unable to exploit the full potential of these data. New paradigms for searching and comparing publicly available microarray results are needed to promote widespread, investigator-driven research on shared data.

To meet this need, we developed and implemented a bioinformatic strategy, termed EXALT (EXpression signature AnaLysis Tool), to enable comparisons of microarray data across experimental platforms, different laboratories, and multiple species. Our system allows investigators to use gene expression signatures (also referred to as gene sets) to query a large formatted collection of microarray results. We accomplished this by first transforming a large collection of gene expression data into a rank ordered format of differentially expressed gene signatures within each experiment. Our strategy avoids the difficulties encountered in direct comparisons of raw microarray observations, and it is not hampered by different experimental platforms. This new approach to mining shared microarray data may have greatest value when it is offered as an online tool for mining data in a repository such as GEO.

## Encoding gene expression signatures

In developing EXALT, we embraced the philosophy that direct comparisons of raw microarray data would be neither feasible nor beneficial. Rather than compare raw data, we chose to implement a search paradigm that matches gene expression signatures deduced from pre-processed (normalized, background subtracted) data, such as that deposited in the GEO database. Because of this feature, EXALT can compare data from any microarray platform and is not dependent on the methods used for the initial data processing. The output from EXALT provides similarity scores and statistical confidence levels for each signature match, thus allowing rapid perusal of relationships between the query data and entries in a database of other microarray experiments.

In order to create a searchable database, we first developed a data structure to encode gene expression signatures that incorporates three attributes, organized into 'triplets', of genes exhibiting significant differences in expression. Each triplet consists of an individual gene identifier, a statistical score, and a direction code indicating whether the gene is expressed at a higher (U for 'upregulated') or lower (D for 'downregulated') level between control and experimental groups. Thus, a gene expression signature, as defined by EXALT, is a set of significant genes with their corresponding statistical scores and direction codes. In essence, a signature (or group of signatures) represents a statistically validated 'fingerprint' associated with a biologic observation made from a gene expression experiment.

A computational pipeline (array expression signature pipeline [AESP]) was implemented to convert automatically microarray data from GEO and other sources into an encoded gene expression signature database (SigDB). For this database, each microarray study was partitioned into three levels: datasets, groups, and samples. EXALT required that each microarray study had one to many datasets based on its experimental design, and that each dataset included at least two groups. In each group, EXALT further required at least two samples to serve as biologic replicates. Each sample described the abundance measurements for each feature element obtained from a single hybridization or experimental condition. Two or more groups were needed to generate statistical comparisons. Significant genes were defined from two groups of samples by calculating a Student's *t*-statistic, significant gene *P *value (false positive rate), and Q value (false discovery rate). Correspondingly, gene expression signatures are collections of significant genes determined from statistical comparisons of groups. Because a microarray study can produce one or many gene expression signatures, depending on the number of groups, we related the maximum total number of signatures (TNS) to the group number (N) in the following equation: TNS = (N × [N - 1])/2.

Among 874 GEO datasets representing microarray experiments performed using human, mouse, or rat tissues, 620 (75%) were successfully converted into gene expression signatures. The extracted signatures (total 16,181; average 1,683 significant genes per signature) from 14,303 hybridizations populated three separate SigDB files for human, mouse, and rat. The signatures in SigDB are designated as subject signatures. Most datasets were either single-channel intensity data, usually corresponding to Affymetrix microarrays, or dual-channel ratio data, usually corresponding to spotted cDNA microarrays. Additional SigDB entries originated from published microarray studies that were not deposited in GEO, as described in the Materials and methods section (below).

## Design and validation of EXALT

**Figure 1:**
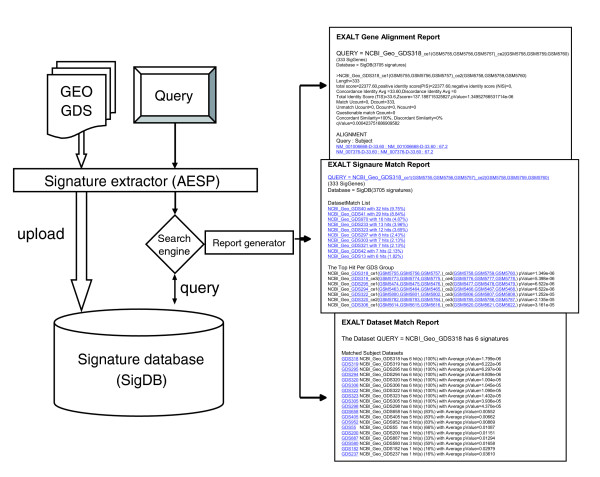
Schematic representation of EXALT system. A data flow diagram of computational steps is shown on the left, illustrating input of subject (Gene Expression Omnibus [GEO] dataset [GDS]) and query datasets, extraction of gene expression signatures, comparison with signature database, and generation of reports. Representative EXALT outputs are illustrated on the right, with three report levels including gene alignment, signature matches, and dataset matches. Reports are coded in HTML and include hypertext links (underlined blue text) to publicly accessible data sources or to other report levels. Some special terms were used in the gene alignment report. 'Total score' is the sum of positive identity score (PIS) and negative identity score (NIS). 'Concordance Identity Avg' is the average PIS per signature gene. 'Discordance Identity Avg' is the average NIS per signature gene. 'Concordant Similarity' is the number of concordant genes expressed as a percentage of the total number of genes in the signature. 'Discordant Similarity' is the number of discordant genes expressed as a percentage of the total number of genes in the signature. 'Alignment' refers to the list of query and matched subject triplets. AESP, array expression signature pipeline; EXALT, EXpression signature AnaLysis Tool; SigDB, signature database.

The EXALT system consists of four components (two program pipelines, SigDB, and search engine) and a web interface (Figure 1). To compare a user-defined query with SigDB, gene expression signatures were first extracted from a pre-processed query data set using AESP. Each user-defined query signature was then compared with every subject signature in SigDB by computing similarity scores and confidence levels. Thus, all signatures from the query dataset were compared with all signatures in SigDB. The EXALT comparisons were based on estimating the degree of signature similarity expressed as a normalized total identity score (TIS) between expression signatures derived from a query dataset and signatures in SigDB (see Materials and methods, below). The significance of the similarity was determined by a simulation analysis (see Materials and methods, below). Finally, reports of similarity were summarized at three levels (gene, signature, and dataset) and sent to the user via an HTML report pipeline (HRP). All results presented here were summarized from dataset-level reports, and the confidence levels are expressed as adjusted mean *P *values (see Materials and methods, below).

As a prerequisite for using EXALT, a user-defined, pre-processed query dataset must be in a simple table format or the GEO simple omnibus format in text (SOFT) format. Then, the user can upload the pre-processed microarray dataset to the EXALT web server [5] by selecting the choice 'Uploading a query dataset' in the top menu bar and obtaining a unique dataset tracking identifier (ID). The EXALT server currently runs query datasets in a batch mode. When analysis is complete, the user can retrieve the EXALT result using the tracking ID. Other features such as searching and browsing signatures from the EXALT databases are under development.

To validate EXALT, we first tested whether the system could correctly identify microarray datasets through signatures that pre-existed in SigDB. We converted 124 randomly selected GEO datasets (GDSs) with AESP and used these to query SigDB. The number of signatures varied from 1 to 777 for these datasets. All 'hits' in the database were ordered by adjusted mean *P *value and the percentage of matching query signatures. Results from this analysis demonstrated that the top 'hits' for each GDS, as defined by lowest adjusted mean *P *value and greatest percentage of matching query signatures, were perfectly concordant with the corresponding entries in SigDB. Twenty representative matching records are presented in Table 1. These results demonstrated that EXALT was able to identify datasets correctly through comparisons between query and subject signatures.

**Table 1:** Self-matching test results for datasets compared using EXALT

Query dataset	Number of query signatures	'Top hit' dataset	Number of 'top hit' signatures (% of query)	*P *value^a^
GDS1011	4	GDS1011	4 (100%)	1.08 × 10^-06^
GDS1023	7	GDS1023	7 (100%)	3.00 × 10^-06^
GDS1030	6	GDS1030	6 (100%)	2.84 × 10^-06^
GDS1048	103	GDS1048	103 (100%)	2.84 × 10^-07^
GDS422	65	GDS422	65 (100%)	2.03 × 10^-07^
GDS430	6	GDS430	6 (100%)	5.55 × 10^-07^
GDS44	10	GDS44	10 (100%)	1.17 × 10^-07^
GDS592	777	GDS592	777 (100%)	1.08 × 10^-06^
GDS666	5	GDS666	5 (100%)	6.84 × 10^-07^
GDS690	2	GDS690	2 (100%)	6.48 × 10^-07^
GDS700	2	GDS700	2 (100%)	1.06 × 10^-06^
GDS711	15	GDS711	15 (100%)	1.45 × 10^-07^
GDS264	6	GDS264	6 (100%)	2.94 × 10^-08^
GDS279	2	GDS279	2 (100%)	1.84 × 10^-06^
GDS286	14	GDS286	14 (100%)	1.04 × 10^-06^
GDS292	6	GDS292	6 (100%)	1.17 × 10^-05^
GDS948	6	GDS948	6 (100%)	8.28 × 10^-06^
GDS955	14	GDS955	14 (100%)	2.51 × 10^-06^
GDS958	2	GDS958	2 (100%)	2.56 × 10^-05^
GDS961	1	GDS961	1 (100%)	2.65 × 10^-07^

^a^Adjusted mean *P *value of all matching datasets. GDS, Gene Expression Omnibus dataset. EXALT, EXpression signature AnaLysis Tool.

## Relationship of statistical with biologic significance

**Figure 2:**
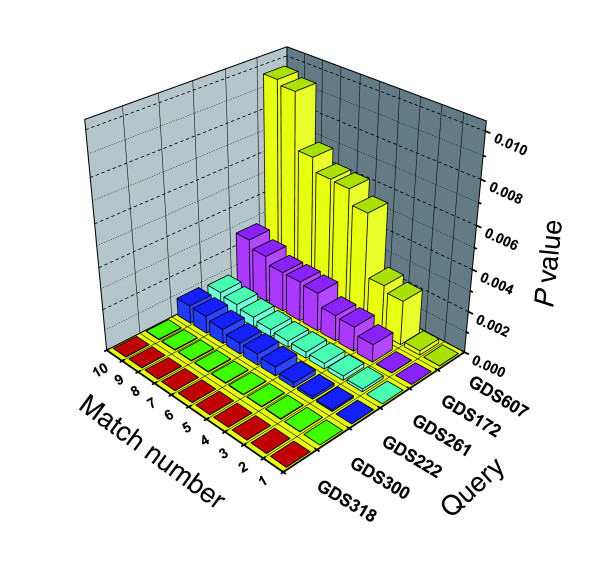
Significance trends among matching datasets. Six representative query datasets from the 124 self-matching results were compared using EXALT with all Gene Expression Omnibus (GEO) records. Corresponding adjusted mean *P *values for each dataset match are plotted on a log_10 _scale. The match number reflects the rank order of adjusted mean *P *values for each query dataset with one representing the best match. Self-matches exhibited the lowest adjusted mean *P *value for all query datasets and were ranked 1 in each search. EXALT, EXpression signature AnaLysis Tool; GDS, GEO dataset.

We next considered whether the output of EXALT could be used to judge the degree of biological relatedness between query and subject datasets. In Figure 2, we plotted the trend in adjusted mean *P *value for the top ten matches for six of the data queries derived from the 124 self-matching results. In each case, the first indexed 'hit' (match number 1) represented a self-match and the other nine were matches with subject datasets having varying levels of similarity.

The variation in adjusted mean *P *value trends among the query datasets illustrated in Figure 2 may potentially represent different biologic relationships between the query and the subject datasets. To explore this idea further, we examined the dataset matches for two specific queries (GDS318 and GDS607) that exhibited marked differences in *P *value trends. For the query GDS318, all ten top 'hits' had adjusted mean *P *values similar to the self-match (*P *< 1.55 × 10^-05^). By contrast, adjusted mean *P *values for subject datasets matching query GDS607 increased steadily through the progression of ordered hits. To determine whether these different adjusted mean *P *value trends reflect different biologic relationships, we explored the annotations for each matching dataset. Matches to GDS318 belong to the same cluster of datasets (anchored by GDS318 set) from a single microarray study [6]. The goal of that study was to examine time-dependent changes in gene expression for mouse splenic B lymphocytes stimulated with 33 different ligands known to directly induce or co-stimulate proliferation. The specific ligand used in generating GDS318 was stromal cell derived factor-1, whereas the ligands studied in the matching subject datasets were secondary lymphoid-organ chemokine, bombesin, B-lymphocyte chemoattractant, terbutaline, insulin-like growth factor-1, tumor necrosis factor-α, 2-methyl-thio-ATP, and sphingosine-1-phosphate. All of these ligands induce similar physiologic events, including B-cell migration and homing [7,8], lymphocyte trafficking [9,10], and mitogenic activation [11]. These results indicate that EXALT can define related gene expression signatures evoked by a heterogenous group of ligands.

By contrast, the annotations for datasets matching GDS607 reflect greater biologic heterogeneity. The GDS607 dataset originated from a study of mouse spermatogenesis and testis development, and nearly half (four out of nine) of the matching subject datasets are biologically related. For example, GDS662, GDS704, GDS606, and GDS660 refer to studies of spermatogenesis and embryonic testis. However, the biologic relationships of GDS607 to the remaining five matching datasets are less clear: GDS900 (kidney inner medulla from aquaporin-1 null and wild-type mice), GDS604 (neurofibromatosis and neurodevelopment), GDS592 (expression profiles from 61 physiologically normal tissues), GDS14 (lung responses to allergic stimuli), and GDS61 (vascular remodeling in pulmonary hypertension). We interpret these findings as an illustration that the level of statistical significance defined by EXALT correlates generally with biologic relatedness among experiments. However, these results may also be informative as to less obvious relationships that will require additional investigations to be fully revealed.

## Cross-platform comparisons of expression signatures

We next tested whether EXALT could identify related gene expression signatures in biologically related datasets generated using different microarray platforms. For this test we utilized publicly available expression data generated from the NCI-60 panel of cancer cell lines by three independent laboratories [12-14] using either oligonucleotide (Affymetrix) or spotted cDNA arrays. Previously, Kuo and coworkers [15] demonstrated that comparisons of primary data from two of these studies revealed poor correlation of individual gene expression levels when the two distinct microarray platforms were compared. They attributed the discordance to probe-specific factors and expressed pessimism about the prospects of comparing data across platforms. However, greater concordance was observed when comparisons were restricted to the subset of genes (generally < 25%) for which there was a high confidence level of identity between the two array platforms [16], or when analyses focused on gene sets sharing similar biologic function or other attributes [17].

We used EXALT to compare expression signatures obtained from analysis of NCI-60 expression data representing nine different cancers (breast, colon, prostate, central nervous system, leukemia, melanoma, lung, renal, and ovarian; Table 2). Expression data from individual cell lines derived from the same cancer type were assumed to represent biologic replicates that were more similar within a group than between different groups. We deduced expression signatures from each study, then added these signatures (*n *= 89) to SigDB to enable EXALT analysis (Table 2). Next, we used the expression signatures as queries to search SigDB, and then ordered the results based on adjusted mean *P *value corresponding to each query dataset. The primary goal of these comparisons was to determine whether similarities of expression signatures among biologically related datasets could be detected across different microarray platforms.

**Table 2:** Datasets from gene expression studies of the NCI-60 cell lines

Dataset name	Array type	Total clones	Cancer classes	Signature number
Stanford_Brown_NatGenetV24P227	cDNA	9,706	9	36
Harvard_Kohane_PNASV97P12182	Affy	7,245	9	30
MIT_Golub_PNASV98P10787	Affy	6,810	9	23

**Figure 3:**
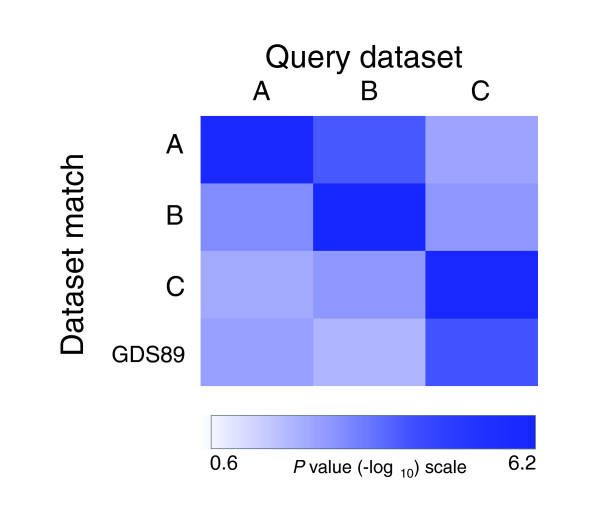
Cross-platform dataset matching revealed by EXALT. Heat-map illustrating the statistical significance among NCI-60 gene expression profiling experiments. The datasets include those reported by Staunton and coworkers [13] (dataset A), Butte and colleagues [12] (dataset B), Ross and coworkers [14] (dataset C), and GDS89. Datasets A and B were generated using Affymetrix arrays, whereas datasets C and GDS89 were generated using spotted cDNA arrays. Dataset comparisons exhibited adjusted mean *P *values below 0.01 (corresponding to values > 2.0 on the -log_10 _scale). The greatest significance levels were observed for self-matching and matches between studies using the same microarray platform. EXALT, EXpression signature AnaLysis Tool; GDS, Gene Expression Omnibus dataset.

Figure 3 illustrates the significance levels for EXALT analyses organized by query dataset. The most significant match for each comparison was a self-match, and the next most closely related signatures were between studies that used the Affymetrix platform (query datasets A and B; Figure 3). Interestingly, EXALT also detected GDS89, an updated full version of dataset C generated by the same research group using spotted cDNA arrays. To test whether other NCI-60 data were present that were not identified by EXALT, we searched the original GEO database (May 2006 release) using various key words, including 'NCI60', and only one additional dataset (GDS88) was identified. GDS88 consisted of four groups representing four different cancer types, but three out of four cancer types had only one sample. Therefore, the experimental design in this dataset could not be used by EXALT to define signatures, and this explains why no match was found to GDS88 in the initial search. Although the greatest significance levels were observed in self-matching datasets and between expression data obtained using the same microarray platform, there was also statistical confidence across platforms for these biologically related data sets (datasets B and C). These findings demonstrate that EXALT can infer biologic relationships between datasets generated using different array platforms.

## Use of EXALT in meta-analysis

Meta-analysis has been demonstrated to provide a strategy for exploiting comparable microarray data from multiple sources to validate observations made by a single study [18]. We tested whether EXALT could enable meta-analysis of microarray data for the purpose of result validation in the setting of cancer gene expression, specifically breast cancer.

We selected a query dataset derived from a published study that examined gene expression differences among 69 estrogen receptor (ER)-negative and 226 ER-positive tumors using inkjet-synthesized oligonucleotide microarrays [19]. The comparison between ER-positive and ER-negative sample groups enabled EXALT to extract one gene expression signature. Using EXALT to search SigDB, we identified a single matching subject dataset (GDS1329; adjusted mean *P *value = 0.0002) obtained from a study using Affymetrix HG-U133A arrays. Interestingly, GDS1329 involved an analysis of 49 breast cancer tumors classified into luminal, basal, and a novel apocrine cell type [20]. Importantly, luminal tumors are typically ER-positive, whereas basal tumors are ER-negative. Three signatures were generated from this study design, and the query signature had a significant and specific match to one of the three. The matching subject signature was derived from a specific comparison between 16 basal ER-negative tumor samples and 27 luminal ER-positive samples. This finding suggests that EXALT successfully validated breast cancer ER-negative versus ER-positive gene expression signatures by comparing two datasets generated by independent groups using different microarray platforms. This result also illustrates how EXALT can be used to identify biologically related datasets on the basis of inherent properties of gene expression signatures.

The use of expression profiles as biomarkers to predict disease prognosis and outcome has become an important adjunct diagnostic tool in cancer [21,22]. However, both training and testing datasets in such predication models typically originate from the same dataset or study. Using an *in silico *validation strategy, such as we have illustrated here with EXALT, the confidence level in identifying predictive signatures could potentially be increased without performing additional experiments.

## Other tools for meta-analysis of microarray data

There are many obstacles to the sharing and widespread use of microarray data. In general, expression measurements made across microarray technologies are not directly comparable [15,23]. Microarray data are inherently more complex than other biologic data types, and there are no universal standards or comparable measurement units. Comparisons among datasets have been particularly difficult [24], as evidenced by the poor correlation between cDNA and oligonucleotide arrays [15,25]. Further advances in genomics and systems biology will require new analysis paradigms that are capable of performing comparisons among experiments that are platform independent.

Previously proposed strategies for comparing multiple microarray datasets can be broadly considered in two categories: direct comparisons of significant gene lists, and indirect comparisons based on gene ontology or other shared biologic knowledge. The most simple direct comparison strategy involves comparing lists of significant genes among related studies and visualizing overlapping genes using Venn diagrams or other methods. Automated versions of this approach such as L2L [26] and LOLA [27], provide quick methods with which to compare lists, but they are quite limited by database scale and the reliance upon potentially heterogeneous analysis strategies used by the original studies from which the lists are generated.

A more advanced comparison strategy of significant gene lists is provided by Oncomine [18], a comprehensive and expertly annotated database of gene expression studies related to cancer. This analytical tool enables searches to identify cancer-related expression data that demonstrate significant differential expression of a single gene of interest or a list of significant genes related to a specific cancer type. Differential expression data are pre-computed in Oncomine using a uniform statistical algorithm, and the developers of this system have demonstrated success in performing comparative meta-profiling to identify shared gene expression signatures across several experiments, although this feature does not appear accessible to the casual user. This system is limited to cancer-related gene expression studies. Another described approach to cross-platform analysis of microarray data, referred to as 'second order analysis', has been applied to deduce networks of transcription factors in yeast [28]. In this approach, the expression patterns of co-expressed gene pairs or 'doublets' were examined across multiple datasets to infer functional linkages (first order analysis). Then, groups of doublets are clustered together based on similar patterns of co-expression. Although capable of elucidating hidden functional linkages among genes, utilization of this method requires substantial informatics expertise.

Recently, Lamb and coworkers [29] described a microrray database search algorithm in an application called the Connectivity Map (CMAP). Like EXALT, CMAP performs microarray signature based comparisons, but the two strategies have several important distinctions. At the database level, CMAP has a focused goal to profile drug-related cancer signatures in ten cell lines, and therefore only a small number of signatures (564) were generated. By contrast, SigDB used by EXALT included 16,181 signatures, representing hundreds of different experimental types from many different tissues. All collected subject signatures in CMAP were derived from one laboratory using a single microarray platform, and signatures derived from other platforms were not demonstrated to work with CMAP. Again, by contrast, SigDB contained data generated with multiple platforms that are fully accessible by EXALT. Other differences include the lack of a unified method for query signature production in CMAP and restrictions on signature length (1,000 genes), whereas EXALT has stringent requirements for query signatures and no limit to the number of genes in a signature (average signature length in SigDB is 1,683 genes). Finally, even though both strategies use signed rank genes as the basis for signatures from a two-group comparison, CMAP does not require biologic replicates in a sample group and no statistical confidence is assigned to each ranked gene, as is done by EXALT.

## Unique features and limitations of EXALT

We developed EXALT to assist researchers wishing to compare the results of multiple gene expression profiling experiments. A key feature of our approach is that it enables comparative analysis of microarray datasets based on signature similarity. A second important attribute is the use of a large, standardized database of microarray data (GEO) and the ability to incorporate virtually any publicly accessible data source. We further implemented an algorithm for performing comprehensive signature comparisons and a user friendly report format. These features provide a potential platform for sharing and comparing all microarray data in a manner suitable for widespread use.

The encoded signatures used by EXALT can serve as unique identifiers for the datasets from which they are derived. Commonly used microarray data analysis methods identify a small fraction of all data based on statistical differences in gene expression. EXALT follows the same principle to extract significant genes from pre-processed microarray datasets, but it further compiles these data into a searchable format. This abstraction process reduces the total amount of data by more than 1,000-fold and allows for a more efficient and accurate search. More importantly, the nonparametric reduction in the volume of data achieves the goal of making different microarray expression datasets comparable. Even though the extracted signatures represent only 10% of the original data records, our results of self-matching (Table 1) indicate that they are unique and sensitive enough to identify original datasets through signature comparisons.

EXALT, like all other methods for analyzing microarray data, has defined limitations. Signatures were not always extractable from microarray datasets. Some GEO records did not have sufficient information to evaluate statistically. Similar to the GEO analysis tool and Oncomine, EXALT uses a single significance test (*t*-test) to extract signatures from all experimental designs, and significant genes were defined based on a two-group comparison strategy. No signature could be produced if a comparison between two groups was not statistically significant. Our method adheres strictly to the group design specified by the investigators, and additional novel comparisons within a dataset are not enabled. Signatures resulting from multiple group comparisons in the original dataset (for instance, time series experiments) could not be analyzed because the current GEO data structure does not provide a computable attribute to identify this type of experiment or hypothesis. However, other statistical comparison methods in conjunction with additional user controls are being considered for future implementations of EXALT. Transcripts (expressed sequence tags) that have not been assigned to known genes having valid RefSeq identifiers cannot be included in signatures, and this will be a limitation until gene nomenclature becomes universally comprehensive and standardized.

## Potential applications of EXALT

There are many potential applications of global microarray data comparisons using EXALT. For example, investigators can gain significant increases in the power of detecting differentially expressed genes [30] through *in silico *validation and comparisons with homologous microarray datasets. EXALT can also enable large-scale searches for 'modules' of signatures with coordinated transcription across a wide range of conditions in three distinct species. Drug discovery is another area driving interest in comparing microarray datasets. Therapeutic effects and toxicity of new drugs could be investigated by correlating gene expression signatures associated with known drug or toxic responses [23,31,32]. Finally, enabling widespread use and comparisons of microarray data will enhance the value of public repositories such as GEO and stimulate other innovative approaches to exploiting these data.

## Materials and methods

### Data collection

We collected publicly available gene expression data from several sources. Our primary source of preprocessed microarray data sets was the GEO [33]. We used the May 2006 release of GEO. The logically related samples from the experiments represented by these records define GEO series records. About one-third of GEO series records have passed GEO internal control processes and are designated as GEO Data Sets (GDSs). GDS records are curated sets of gene expression measurements with processes such as background correction and normalization that are consistent across datasets [3]. A GDS record represents a collection of biologically and statistically comparable GEO samples that can be examined using the GEO suite of data display and analysis tools. Other datasets, as described below, were downloaded from publicly accessible sites named by the following conventions: company or institution; last name of corresponding author or dataset name; and journal abbreviation, volume (starting with 'V'), and starting page number (starting with 'P').

The NCI-60 datasets were derived from 60 human cancer cell lines and used by the US National Cancer Institute to screen for new antineoplastic drugs [15,16]. The NCI-60 panel used in this study included cell lines derived from breast, colon, prostate, and central nervous system cancers, and leukemia. The NCI-60 cell lines had been profiled using cDNA micorarrays (Stanford_Brown_NatGenetV24P227) [14], and Affymetrix oligonucleotide HU6800 microarrays (Harvard_Kohane_ PNASV97P12182 and MIT_Golub_PNASV98P10787) [12,13]. Additional information is provided in Table 2.

### Extracting gene expression signatures

We developed a four-step process to extract gene expression signatures from a dataset. First, data were formatted, if necessary, to a common data type, namely the SOFT format used by GEO. Reformatting included a minor reconfiguration of data annotation. All gene probe identifiers were translated to the corresponding NCBI Reference Sequence identifiers (RefSeq ID) [34] using our previously described Gene Annotation Project (GAP) database [35]. The RefSeq collection provides a comprehensive, integrated, nonredundant set of gene identifiers. Furthermore, RefSeq IDs are more stable and reliable than UniGene clusters [36]. Second, for every two groups of samples in a dataset, we generated an expression signature. Following file conversion, each gene was assessed for the significance of differential expression using a two-sided Student's *t*-test. When multiple probes have the same RefSeq identifier, we analyzed them separately through the statistical testing step and then grouped them into a single record having a mean *P *value derived from the individual probes. To account for multiple hypothesis testing, *P *values determined for each significant gene were further adjusted by the false discovery rate (FDR) method using Q values [37]. Third, a list of significant genes with Q value of 0.2 or less was generated. For each significant gene, a Q score was calculated as the logarithm of reciprocal Q value (-log [Q value]). Finally, a gene expression signature was generated as a list of 'triplets', each defined as RefSeq ID - direction code - Q score. The direction code is defined by the relative difference between two group means and can have one of three values (U [up], D [down], or X [uncertain]). The order of the two groups is arbitrary, and so the direction code will be reversed if the group order is flipped. However, the approach used to perform signature comparisons (see below) is not affected by the order of groups assigned at the time when signatures were extracted. Signatures were stored in a flat file database (SigDB).

Queries to our system are facilitated through a web-based computational pipeline (AESP) to automate the extraction of gene expression signatures from microarray datasets [5]. Input information includes dataset name, sample number, sample names, microarray platform, and group assignments. AESP performs translation of probe IDs, significance tests, and the encoding of gene expression signatures for use by EXALT. A unique dataset tracking ID is assigned to each input query dataset that can be used later to retrieve an EXALT report.

The EXALT server was implemented on a high throughput multi-CPU Linux cluster using PERL and system scripts. The primary platform was the Vanderbilt University Advanced Computing Center for Research & Education (ACCRE), which currently consists of 1,302 processors in 651 nodes, each with at least 1 gigabyte of memory, and dual gigabit ethernet ports. Processing all available GDS records (874 records, approximately 2 gigabytes in size) from 14,303 hybridizations on a 35-CPU ACCRE subcluster required an average of 72 hours CPU time. A typical query dataset contains three groups. It can generate three signatures, with about 1,000 signature genes per signature. A typical EXALT analysis (for instance, production of signatures and then comparison with SigDB) will take approximately 2 hours on a single CPU.

### Comparison of gene expression signatures

In an EXALT analysis, each query signature was compared with every subject signature in SigDB. For each pair of query and subject signatures with lengths L_q _and L_s _, a total identity score (TIS) was computed in three steps. First, the signatures were aligned by matching RefSeq ID, then the direction codes (U, D, or X) for matching genes were determined to be concordant (U-U or D-D), discordant (U-D), or uncertain (direction code X in either query or subject). Next, the Q scores were summed separately for concordant and discordant matches to give a positive identity score (PIS) and a negative identity score (NIS), respectively, using the following equations:

$$\text{PIS}=\sum_{\text{i}=1}^{\text{N}}\left({\text{S}}_{\text{iq}}+{\text{S}}_{\text{is}}\right)$$

$$\text{NIS}=-\sum_{\text{j}=1}^{\text{M}}\left({\text{S}}_{\text{jq}}+{\text{S}}_{\text{js}}\right)$$

Where N and M are numbers of concordant and discordant matches, respectively, and S_iq _and S_is _(S_jq _and S_js _) are Q scores for the i-th concordant (j-th discordant) match in the query and subject signatures. The NIS score was assigned a negative value because of its opposite direction from PIS scores. Matches with at least one direction code of X and all nonmatching genes were excluded from the identity score calculations. Finally, the TIS was computed as the absolute value of the sum of PIS and NIS divided by the sum of signature lengths (L_q _+ L_s _) using the following: TIS = |PIS + NIS|/(L_q _+ L_s _).

### Defining significance level

We carried out simulations to determine the statistical significance of TIS values. We generated 1,000 random query signatures and computed TIS between each query signature and each subject signature in SigDB. The random query signatures had similar properties (length distribution, RefSeq ID frequency, and uniqueness) as compared with those of the actual data. The results suggested that TIS score correlated with query signature length. To adjust for the influence of query signature length, we derived the mean and standard deviation (SD) of TIS as functions of query length and then normalized TIS by converting to Z score using the following equation: Z_TIS _score = (TIS - mean)/SD, where mean and SD are functions of query length. This enabled us to generate an empirical distribution of Z_TIS _scores. For a real query, we followed the same procedure to calculate the Z_TIS _score, and compared it with the empirical distribution to estimate corresponding query *P *value. A query is statistically significant if its *P *value is 0.01 or less.

### Reporting EXALT results

An algorithm for reporting EXALT results was implemented that considers information at three different levels: dataset, individual expression signature, and significant genes within a signature. The gene-level report contains alignments of significant gene triplets that were matched from within a pair of query and subject signatures. The signature level report contains matches between whole expression signatures. A query signature may have none to many significant matches or 'hits' in a subject signature in SigDB, and the match with the smallest query *P *value is designated as the 'top hit'.

The most global comparison is a dataset (top-level) report, which describes the similarity between a query dataset and a dataset in SigDB. For this, a query dataset may have one or more query signatures, and each query signature may match one top hit in each subject dataset. The most similar dataset is selected based on two criteria. The first criterion is the average of query *P *values from all top hit signatures divided by the total number of top hits (TS). The second criterion is the top hit ratio calculated as the total number of top hits divided by the total number of query signatures (TQ). An adjusted mean *P *value is calculated by an arithmetic average of all top hit query *P *values divided by the top hit ratio, and this is used to rank the confidence levels of data set matches.
